# Clinical and Biological Predictors of Plasma Levels of Soluble RAGE in Critically Ill Patients: Secondary Analysis of a Prospective Multicenter Observational Study

**DOI:** 10.1155/2018/7849675

**Published:** 2018-05-10

**Authors:** Thibaut Pranal, Bruno Pereira, Pauline Berthelin, Laurence Roszyk, Thomas Godet, Russell Chabanne, Nathanael Eisenmann, Alexandre Lautrette, Corinne Belville, Raiko Blondonnet, Sophie Cayot, Thierry Gillart, Yvan Skrzypczak, Bertrand Souweine, Damien Bouvier, Loic Blanchon, Vincent Sapin, Jean-Michel Constantin, Matthieu Jabaudon

**Affiliations:** ^1^Department of Perioperative Medicine, CHU Clermont-Ferrand, Clermont-Ferrand, France; ^2^Department of Clinical Research and Innovation (DRCI), CHU Clermont-Ferrand, Clermont-Ferrand, France; ^3^Université Clermont Auvergne, CNRS UMR 6293, and INSERM U1103, GReD, Clermont-Ferrand, France; ^4^Department of Medical Biochemistry and Molecular Biology, CHU Clermont-Ferrand, Clermont-Ferrand, France; ^5^Intensive Care Unit, Jean Perrin Comprehensive Cancer Center, Clermont-Ferrand, France; ^6^Medical Intensive Care Unit, UMR CNRS 6023, CHU Clermont-Ferrand, Clermont-Ferrand, France

## Abstract

**Rationale:**

Although soluble forms of the receptor for advanced glycation end products (RAGE) have been recently proposed as biomarkers in multiple acute or chronic diseases, few studies evaluated the influence of usual clinical and biological parameters, or of patient characteristics and comorbidities, on circulating levels of soluble RAGE in the intensive care unit (ICU) setting.

**Objectives:**

To determine, among clinical and biological parameters that are usually recorded upon ICU admission, which variables, if any, could be associated with plasma levels of soluble RAGE.

**Methods:**

Data for this ancillary study were prospectively obtained from adult patients with at least one ARDS risk factor upon ICU admission enrolled in a large multicenter observational study. At ICU admission, plasma levels of total soluble RAGE (sRAGE) and endogenous secretory (es)RAGE were measured by duplicate ELISA and baseline patient characteristics, comorbidities, and usual clinical and biological indices were recorded. After univariate analyses, significant variables were used in multivariate, multidimensional analyses.

**Measurements and Main Results:**

294 patients were included in this ancillary study, among whom 62% were admitted for medical reasons, including septic shock (11%), coma (11%), and pneumonia (6%). Although some variables were associated with plasma levels of RAGE soluble forms in univariate analysis, multidimensional analyses showed no significant association between admission parameters and baseline plasma sRAGE or esRAGE.

**Conclusions:**

We found no obvious association between circulating levels of soluble RAGE and clinical and biological indices that are usually recorded upon ICU admission. This trial is registered with NCT02070536.

## 1. Introduction

The receptor for advanced glycation end products (RAGE) is a multiligand receptor from the immunoglobulin superfamily of cell surface receptors [1, 2]. Most RAGE ligands are implicated in inflammation and cell migration processes, and indeed, the expression of RAGE is low under physiological settings but can be upregulated in response to inflammation [3]. Upon RAGE stimulation, the transcription factor NF-*κ*B is translocated to the nucleus and binds to the promoter region of RAGE which enhances RAGE mRNA translation itself [4]. RAGE has therefore the potential to function as a master switch capable of converting a transient proinflammatory response, evoked by an inflammatory stimulus into sustained cellular dysfunction [5, 6].

In addition to the full-length receptor, RAGE undergoes extensive alternative splicing to produce a variety of transcripts with diverse functions. Major isoforms are the full-length mRAGE, a secreted form RAGE v1 (also named as endogenous secretory (es)RAGE) and an N-terminally truncated isoform RAGE v2 (previously named N-truncated RAGE). However, endogenous soluble RAGE may also be generated by mechanisms other than alternative splicing, such as membrane-associated proteases, including the sheddase A disintegrin and metalloprotease-10 (ADAM-10) and the matrix metalloproteinase-9 (MMP-9) [7–9]. Therefore, the total pool of soluble (s)RAGE is mainly composed of cleaved (c)RAGE and esRAGE isoforms. Soluble RAGE may function as a decoy for ligands, thus preventing the interaction with the membrane-anchored full-length RAGE [10]. Although the roles and regulation pathways of sRAGE are not fully understood, its circulating levels may inversely correlate with RAGE activity. The abnormal upregulation and activation of RAGEs and thereby inflammatory and immune responses are involved in the development of a number of human illnesses including diabetes, cardiovascular diseases, ischemic injury, osteoarthritis, cancer, Alzheimer's disease, and other inflammatory states [3, 11].

In the settings of intensive care and perioperative medicine, in which many of these diseases may interfere with acute illness, sRAGE is now considered a marker of lung epithelial injury [12, 13]. There is growing evidence supporting a pivotal role for RAGE in the pathophysiology of acute respiratory distress syndrome (ARDS) [14] through the initiation and perpetuation of inflammatory and immune responses, but the roles of the RAGE pathway during lung injury and repair remain incompletely understood to date [15, 16]. Notably, sRAGE has most features of a validated biomarker that could be used in clinical medicine [17], with values for ARDS diagnosis [13, 18–20], assessment of severity, impaired alveolar fluid clearance, and prognosis [13, 18, 19, 21–23], monitoring the response to therapy [24–26] and possibly identifying subgroups (or phenotypes) of patients that could benefit from tailored therapy [23, 27]. However, more validation studies are still warranted prior to its use in clinical research practice.

The goal of this secondary analysis of prospectively acquired data was to investigate, among critically ill patients at risk of developing ARDS, the relationships between plasma levels of RAGE soluble forms (sRAGE, esRAGE) and clinical, biological variables, including common patient characteristics, coexisting conditions, and treatment.

## 2. Methods

### 2.1. Participants

Data used in this ancillary study were prospectively obtained from patients previously enrolled in a large multicenter observational study on the predictive values of RAGE isoforms and gene variants for the onset of ARDS (PrediRAGE study, clinicaltrials.gov identifier: NCT02070536) [28]. Between June 2014 and January 2015, 500 critically ill adult patients admitted to five intensive care units (ICUs) were enrolled in the primary study if they had at least one ARDS risk factor [14]. Patients were excluded if they were admitted for an isolated neurological or neurosurgical diagnosis without any significant medical comorbidities [29]. Patients who met the criteria for ARDS at initial assessment, or within the subsequent 24 hours, were excluded from analysis to ensure removal of ARDS that was present at baseline. Briefly, the main results from the PrediRAGE study indicate that elevated plasma sRAGE and the presence of single nucleotide polymorphism rs2070600 within AGER gene were useful to identify patients at the highest risk of developing ARDS [28].

Patients for whom full biological data set was not available at baseline were excluded from this secondary analysis. The following parameters were collected at baseline: patient characteristics (age, sex, and body mass index) and comorbidities, severity of illness as assessed by the simplified acute physiology score (SAPS II) [30, 31], respiratory and hemodynamic parameters, the ratio of the partial pressure of arterial oxygen to the fraction of inspired oxygen (PaO_2_/FiO_2_), the partial pressure of carbon dioxide (PaCO_2_) and other biological variables (e.g., arterial pH, serum lactate, serum bicarbonate, serum creatinine, leukocyte, and platelets counts), concurrently administered treatments (e.g., corticosteroids and vasopressors), and the lung injury prediction score (LIPS), that is, a clinical prediction score developed with the goal of identifying patients at a high risk for the development of ARDS [32].

Our Institutional Review Board approved the research protocols for the primary study and this ancillary study (*Comité de Protection des Personnes Sud Est VI*, approval number AU1073). All participants, or their next of kin, provided written consent to participate. There was no deviation from the approved protocols.

### 2.2. Primary Outcomes

Primary outcomes of this ancillary study were plasma levels of sRAGE and esRAGE, as measured by duplicate ELISA (R&D Systems, Minneapolis, MN, USA, and B-Bridge International Inc., Santa Clara, CA, USA, resp.) upon ICU admission.

### 2.3. Statistical Methods

Patient characteristics are presented as the mean (±standard deviation (SD)) or median [interquartile range (IQR)] values for continuous data (assumption of normality assessed using the Shapiro–Wilk test) and as the number of patients and associated percentages for categorical parameters. Comparisons between independent groups were performed using the chi-squared or Fisher exact tests for categorical variables and Student *t*-test or Mann–Whitney *U* test for quantitative parameters (normality, assumption of homoscedasticity studied using the Fisher-Snedecor test). Univariate correlations were assessed using Spearman correlation coefficients for quantitative outcomes and with Cohen's coefficients of effect sizes for qualitative variables. As proposed by some authors, we did not apply any mathematical correction to assess type I error [33]. Instead, specific attention was given to the magnitude of improvement and to clinical relevance. A center effect was taken into account as a random effect, when appropriate.

Multivariate adjustments using multidimensional analyses (principal component and multiple correspondence analyses) were performed in order to assess relationships between the modalities of variables and to identify distinct patterns of variables, that is, clusters of variables in which groups of patients have similar characteristics.

Tests were two-sided, with a type I error set at *α* = 0.05. Statistical analyses were performed using Stata 14 (StataCorp LP, College Station, TX, USA) and R version 3.3.1 (R Foundation for Statistical Computing, Vienna, Austria).

## 3. Results

### 3.1. Baseline Characteristics

The baseline characteristics of the study sample are described in Table 1. A total of 294 patients were included in this ancillary study. Most patients (62%) were admitted to the ICU for medical conditions, including septic shock (11%), coma (11%), and pneumonia (9%). Sixty-six percent were male, with a mean age of 61.4 ± 16 years. Most frequent coexisting comorbidities included hypertension (40%), current tobacco smoking (24%), dyslipidemia (21%), atherosclerosis (18%), and diabetes (18%). Primary ARDS risk factors were high-risk surgery (37%), shock (28%), sepsis (23%), and pneumonia (13%). The mean lung injury prediction score was 5 ± 2.5, and baseline SAPS II was 44.3 ± 19.7. 150 (51%) patients were spontaneously breathing, while 43% were treated with invasive ventilation and 6% with noninvasive ventilation.

### 3.2. Clinical and Biological Variables at ICU Admission

Clinical and biological parameters are reported in Table 2 for the whole cohort of patients who were included in this study. In mechanically ventilated patients, median PaO_2_/FiO_2_ was 250 [154–300], with an inspiratory plateau pressure of 17 [14–20] cmH_2_O and positive end-expiratory pressure (PEEP) set at 7 [6–8] cmH_2_O. Tidal volume was 7.4 [6.7–8.1] mL·kg^−1^ of predicted body weight. C-reactive protein at admission was 108 [35.5–182] mg·L^−1^. Baseline plasma levels of sRAGE and esRAGE were 1006 [638–1993] and 470 [202–1000] pg·L^−1^, respectively.

### 3.3. Univariate Analyses of Quantitative Variables

**Figure 1:**
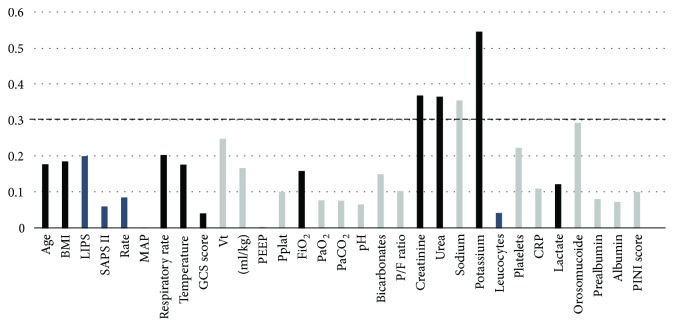
Spearman rho coefficients of the correlation between quantitative variables and plasma sRAGE at ICU admission. Dark bars represent positive correlations; grey bars represent negative correlations. The body mass index (BMI) is the weight in kilograms divided by the square of the height in meters. LIPS: lung injury prediction score; SAPS II: simplified acute physiology score II; MAP: mean arterial pressure; GCS: Glasgow coma scale; Vt: tidal volume; PEEP: positive end-expiratory pressure; Pplat: inspiratory plateau pressure; FiO_2_: fraction of inspired oxygen; PaO_2_: arterial partial pressure of oxygen; PaCO_2_: arterial partial pressure of carbon dioxide; P/F ratio: PaO_2_/FiO_2_; CRP: C-reactive protein; PINI: prognostic nutritional and inflammatory index; ICU: intensive care unit; sRAGE: soluble form of the receptor for advanced glycation end products.

Correlations between sRAGE levels and other quantitative variables were analyzed using Spearman correlation coefficients. Spearman coefficients of correlation (rho) are reported in Figure 1. Weak-to-moderate associations were considered when coefficients were above 0.3. Serum creatinine, urea, and potassium had weak positive correlations with plasma sRAGE, while serum sodium correlated negatively with baseline sRAGE. No other quantitative variable was associated with levels of sRAGE in univariate analysis.

**Figure 2:**
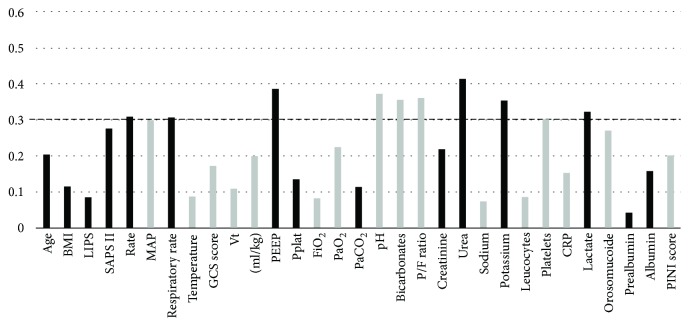
Spearman rho coefficients of correlation between quantitative variables and plasma esRAGE at ICU admission. Dark bars represent positive correlations; grey bars represent negative correlations. The body mass index (BMI) is the weight in kilograms divided by the square of the height in meters. LIPS: lung injury prediction score; SAPS II: simplified acute physiology score II; MAP: mean arterial pressure; GCS: Glasgow coma scale; Vt: tidal volume; PEEP: positive end-expiratory pressure; Pplat: inspiratory plateau pressure; FiO_2_: fraction of inspired oxygen; PaO_2_: arterial partial pressure of oxygen; PaCO_2_: arterial partial pressure of carbon dioxide; P/F ratio: PaO_2_/FiO_2_; CRP: C-reactive protein; PINI: prognostic nutritional and inflammatory index; ICU: intensive care unit; ICU: intensive care unit; esRAGE: endogenous secretory form of the receptor for advanced glycation end products.

Baseline severity of illness (as assessed by SAPS II), heart rate, respiratory rate, PEEP, serum urea, potassium, and lactate correlated positively, albeit modestly, with plasma levels of esRAGE (Figure 2). Arterial pH, serum bicarbonate, and PaO_2_/FiO_2_ negatively correlated with plasma esRAGE. No other quantitative variable was associated with plasma esRAGE in univariate analysis.

### 3.4. Univariate Analyses of Qualitative Variables

**Figure 3:**
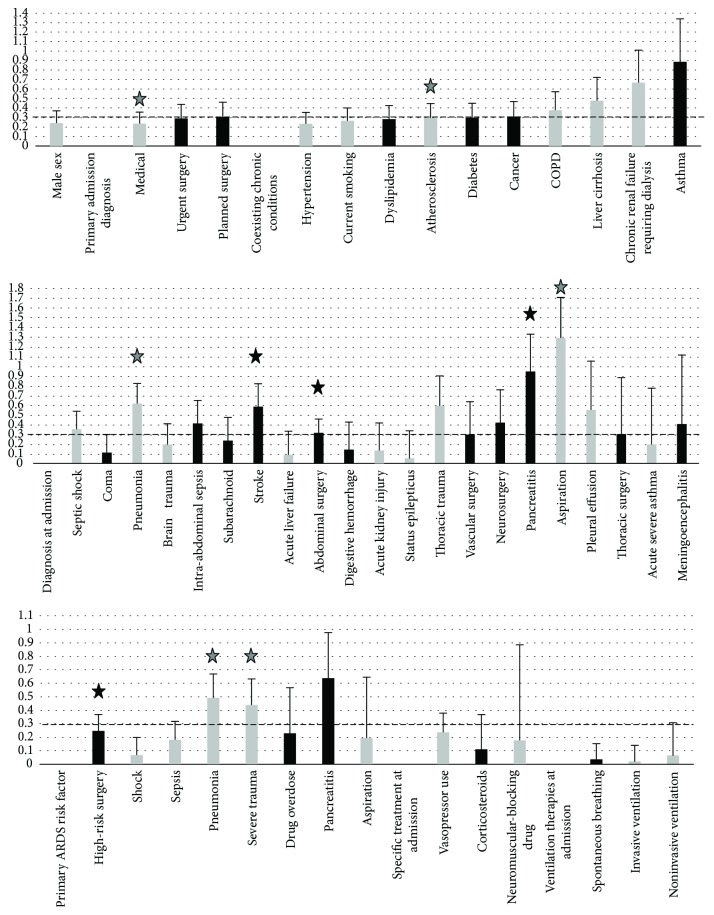
Size effect coefficient (Cohen's *d* coefficient), with upper standard deviation (SD) for baseline qualitative variables tested against plasma sRAGE at ICU admission. Dark bars represent positive values for size effect, grey bars represent negative values, and stars represent coefficient values with 95% confidence intervals that exclude 0, i.e. statistical significance. sRAGE: soluble form of the receptor for advanced glycation end products. ICU: intensive care unit. COPD: chronic obstructive pulmonary disease.

**Figure 4:**
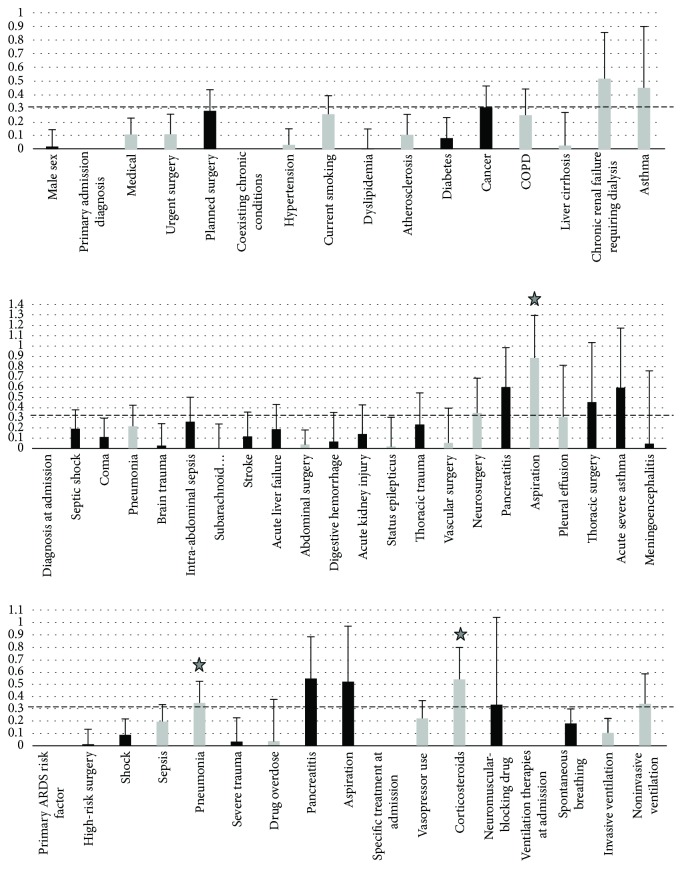
Size effect coefficient (Cohen's *d* coefficient), with upper standard deviation (SD) for baseline qualitative variables tested against plasma esRAGE at ICU admission. Dark bars represent positive values for size effect, grey bars represent negative values, and stars represent coefficient values with 95% confidence intervals that exclude 0, that is, statistical significance. esRAGE: endogenous secretory form of the receptor for advanced glycation end products; ICU: intensive care unit; COPD: chronic obstructive pulmonary disease.

Qualitative variables were analyzed based on effect size calculations. Figures 3 and 4 summarize Cohen's *d* coefficients of effect sizes (with SD) of qualitative variables when tested against plasma sRAGE and esRAGE, respectively. Weak-to-moderate associations were again considered when coefficients were above 0.3.

When tested against plasma sRAGE, variables that had effect size coefficients above 0.3 and 95% CI excluding 0 (i.e., variables with significant associations) were as follows:1 a medical history of atherosclerosis or of chronic renal failure requiring dialysis, ICU admission for medical reasons in general, and ICU admission for pneumonia, stroke, pancreatitis and aspiration, and severe trauma, after high-risk surgery and after abdominal surgery. Such variables were all negatively associated with plasma sRAGE, except stroke, pancreatitis, high risk, and abdominal surgeries (Figure 3).

Variables that were significantly, and negatively, associated with plasma esRAGE were baseline ongoing treatment with corticosteroids and ICU admission for aspiration or pneumonia (Figure 4).

### 3.5. Multidimensional Analyses

Next, we performed multidimensional analyses in order to identify selected variables (i.e., significant in univariate analyses) that best correlated with baseline plasma sRAGE or esRAGE levels. The main objective was therefore to compute two-dimensional graphs with multivariate datasets of variables and then to verify whether such datasets were associated with plasma levels of RAGE soluble forms.

**Figure 5:**
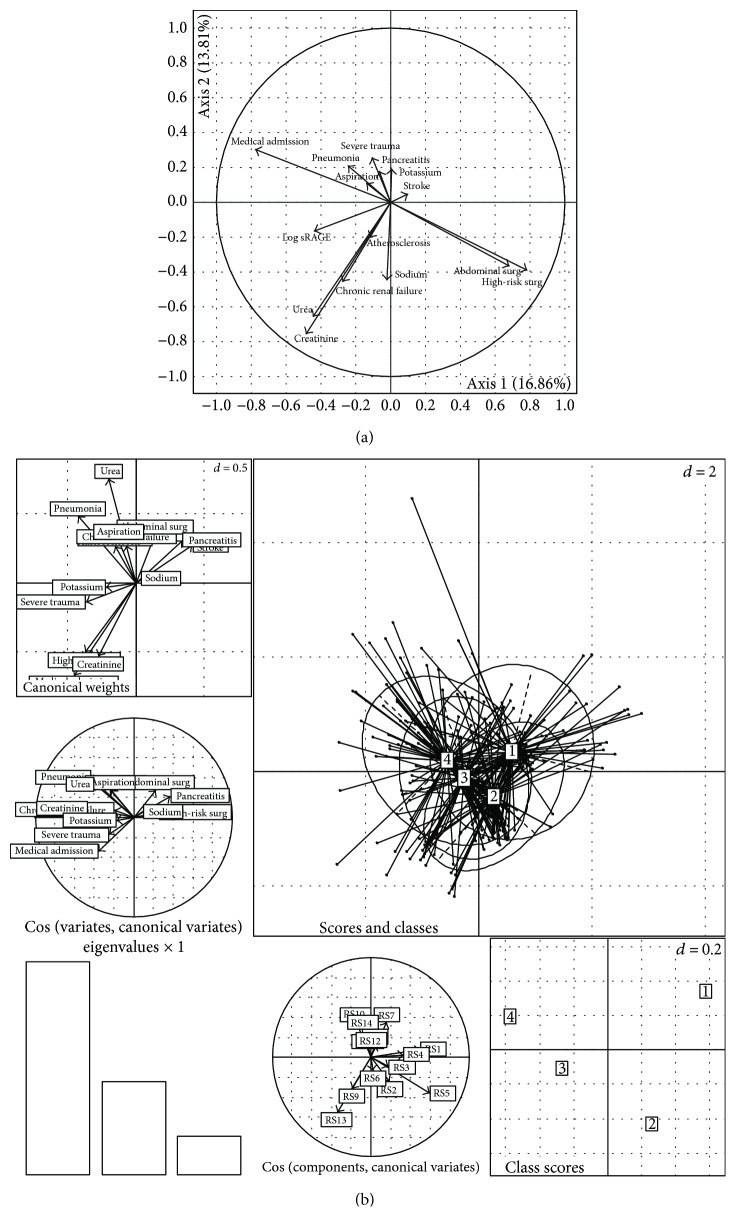
Multidimensional analyses. (a) Principal component analysis (PCA) of clinical and biological variables (significant in univariate analysis) against log-transformed plasma sRAGE. The first two axes of PCA were chosen, as they represent 31% of total inertia and because including other axes did not influence the results (data not shown). PCA suggests an absence of correlation between plasma sRAGE and selected variables. (b) Multiple correspondence analysis (MCA) with mixed quantitative and qualitative variables against plasma sRAGE as subdivided into quartiles. No significant association between quartiles of sRAGE and selected variables was found. [1], [2], [3], and [4] represent the 1st, 2nd, 3rd, and 4th quartiles, respectively. sRAGE: soluble form of the receptor for advanced glycation end products.

We first included selected variables (based on the results from univariate analysis) against log-transformed plasma sRAGE in a principal component analysis (PCA) (Figure 5(a)). The first two axes of PCA were chosen as they represent 31% of total inertia and because including other axes did not influence the results (data not shown). Based on the horizontal axis, patients admitted after abdominal or high-risk surgery were well discriminated from medical patients and those admitted with aspiration, pneumonia, or pancreatitis. The most obvious result from this PCA is that baseline log-transformed sRAGE seems rather well isolated from other variables, thus suggesting an absence of correlation with selected variables. However, the vector for sRAGE is directed in the same way than the vector for kidney-related variables (such as urea, serum creatinine, and a history of chronic renal failure), although these variables may exhibit multicollinearity. Such an association with kidney-related variables was not confirmed when sRAGE was considered an independent variable in PCA.

We also performed another multidimensional analysis (multiple correspondence analysis (MCA)) using quartiles of plasma sRAGE instead of using its log-transformed values (Figure 5(b)). Again, sRAGE was not obviously associated with other selected variables. However, lowest values of sRAGE (i.e., 1st quartile) were separated from those of other quartiles and were slightly, yet insignificantly, related to pancreatitis and stroke as admission diagnoses.

**Figure 6:**
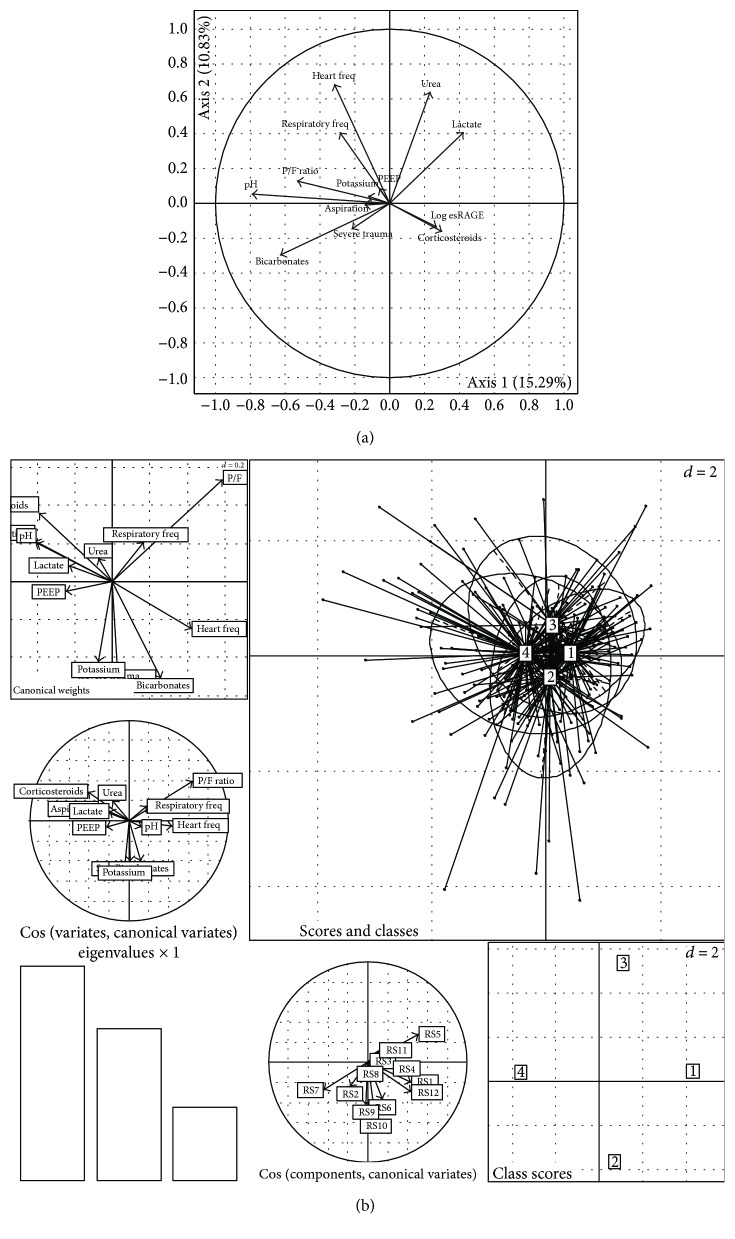
Multidimensional analyses. (a) Principal component analysis (PCA) of clinical and biological variables (significant in univariate analysis) against log-transformed plasma esRAGE. The first two axes of PCA were chosen as they represent 26% of total inertia, and because including other axes did not influence the results (data not shown). PCA suggests an absence of correlation between plasma esRAGE and selected variables. (b) Multiple correspondence analysis (MCA) with mixed quantitative and qualitative variables against plasma esRAGE as subdivided into quartiles. No significant association between quartiles of esRAGE and selected variables was found. [1], [2], [3], and [4] represent the 1st, 2nd, 3rd, and 4th quartiles, respectively. esRAGE: endogenous secretory form of the receptor for advanced glycation end products.

Using the same methodology, we performed PCA against log-transformed plasma esRAGE (Figure 6(a)), with the first two axes representing 26% of total inertia. As against sRAGE, including other axes did not influence the results (data not shown). Results showed great heterogeneity, suggesting the absence of associations between esRAGE and selected variables, except for ongoing treatment with corticosteroids. However, only 2 patients from our cohort received such treatment upon ICU admission, thus supporting statistical insignificance in PCA. This association was not found when esRAGE was computed as an independent variable.

Finally, we performed multidimensional analysis using quartiles of plasma sRAGE instead of its log-transformed values (Figure 6(b)) that confirmed the absence of an association between quartiles of esRAGE and other variables.

## 4. Discussion

In this secondary analysis of prospectively acquired data in a sample of 294 critically ill patients, we aimed at exploring putative associations between plasma levels of RAGE soluble forms (sRAGE, esRAGE) upon ICU admission and baseline demographics, clinical, biological, or therapeutic parameters. All patients from our cohort had at least one identified risk factors of ARDS, and most of them were admitted to the ICU for medical reasons (e.g., septic shock, pneumonia, and coma).

After multivariate adjustment, no significant association was found in this cohort between sRAGE, or esRAGE, and patient characteristics, clinical/biological indices, or therapeutic measures at admission. In univariate analyses, ICU admission for stroke and pancreatitis and after high risk (e.g., abdominal) surgery, as well as high plasma creatinine, urea, and potassium, were factors that were most associated with higher baseline plasma sRAGE, whereas patients admitted to the ICU with pneumonia, aspiration, severe trauma, a medical history of atherosclerosis, and higher plasma sodium levels were more likely to have lower plasma sRAGE. However, such associations were insignificant after multidimensional analysis, thus suggesting no evident association between baseline plasma sRAGE and the main clinical or biological variables that were tested in this study. Admission for pneumonia or aspiration, ongoing treatment with corticosteroids, and higher baseline PaO_2_/FiO_2_, arterial pH or serum bicarbonate were factors that were associated with lower plasma esRAGE in univariate analyses, whereas higher baseline plasma levels of esRAGE were found in patients with higher SAPS II severity scores, higher heart and respiratory rates, higher levels of PEEP, or higher serum urea, potassium, and lactate. Again, however, multidimensional analyses did not reveal a significant interaction between these factors and baseline plasma esRAGE.

The ligation of RAGE is considered a double-edged sword, which may not only play an important role in resolving the pathogenesis of an offending insult but also lead to tissue destruction [34]. In particular, the balance between isoforms of RAGE has been described as an important regulator of RAGE activation both under physiological conditions and in diseases [2, 35]. Generally, membrane-bound (m)RAGE is thought to promote disease pathogenesis and injury by activating the NF-*κ*B pathway. In contrast, sRAGE is thought to be protective by preventing mRAGE signaling in diseases such as tumor growth, metastasis, and diabetic wound healing [36–40]. For example, RAGE expression is upregulated in lung epithelial cells during ARDS; sRAGE (including esRAGE) is secreted into the BAL and is detectable in the serum, serving as a biomarker for the degree of lung injury and acting as a decoy receptor to downregulate the injurious pulmonary inflammatory response [22, 41, 42]. In addition, circulating RAGE soluble isoforms are modified differentially in patients with ARDS, with decreased levels of esRAGE (produced through alternate splicing [43]) and increased sRAGE, mainly through mRAGE cleavage by upregulated matrix metalloproteinases [9, 44], thus illustrating the complexity of the regulation of the RAGE pathway during acute illness.

Many studies have focused on the roles of the RAGE pathway in the development of diseases, in which RAGE soluble forms sRAGE and esRAGE were investigated as biomarkers and/or surrogates for prognosis. Significant associations that were found in our univariate analyses are consistent with previous reports, as they could emphasize the roles of the RAGE pathway in infectious conditions and kidney, lung, and vascular diseases. To our knowledge, our study is the first to focus on predictors of sRAGE and esRAGE in a population of critically ill patients, although previous studies have reported associations between acute illness and RAGE soluble forms. In particular, there are some discrepancies between findings from studies of circulating levels of sRAGE during sepsis: although plasma RAGE may be higher in patients with severe sepsis or septic shock than in nonseptic patients [45], sRAGE might be more of a marker of lung injury during sepsis than a marker of sepsis itself [19]. Indeed, plasma sRAGE was also higher in patients developing ARDS, regardless of the presence or absence of severe sepsis, and correlated with lung injury severity, impaired alveolar fluid clearance, and prognosis [13, 18, 19, 22, 23, 46]. In another inflammatory condition such as acute pancreatitis, higher plasma sRAGE was associated with more severe forms of the disease, but whether the time between pancreatitis onset and sample collection may have influenced these results remains unclear [47]. Interestingly, we found no association of plasma levels of sRAGE or esRAGE with indices of inflammation (e.g., CRP, orosomucoid) and of nutritional status (e.g., prealbumin and albumin) or with the prognostic nutritional and inflammatory index (PINI) in our study, although previous studies have suggested relationships between nutrition, for example, with high intakes of dietary AGEs, and the activation of the RAGE pathway [48–50].

Elevated on-admission sRAGE was associated with an increased risk of developing kidney failure in surgical ICU patients [51], an association that had previously been described in a noncritically ill, nondiabetic, hypertensive population [52]. Such findings may explain, at least partially, why a medical history of chronic renal failure or levels of urea and creatinine had a mild correlation with plasma sRAGE after univariate analysis in our study. In other studies, circulating sRAGE was an independent predictor of cardiovascular events in patients with type 2 diabetes, with contradictory results on whether higher plasma sRAGE was more likely to be associated with stroke and/or with coronary heart disease [53, 54]. In addition, elevated plasma sRAGE was associated with plaque vulnerability in patients with acute myocardial infarction [55]. Probably in line with these results, we found a weak correlation between stroke, as a reason for ICU admission, and sRAGE levels after univariate analysis in our study, but this association was not confirmed after multidimensional analysis. In our cohort, a medical history of diabetes was not predictive of plasma sRAGE or esRAGE. There has been extensive research on sRAGE and esRAGE in patients with type 2 diabetes, and lower levels of both sRAGE and esRAGE may be markers of poor glycemic control [56] and of increased risk of microvascular complications [57]. However, whether elevated sRAGE is associated with a higher or lower risk of cardiovascular risk during diabetes remains controversial [53, 56], and contradictory findings from previous studies may be explained by rather small sample sizes that may have hampered the detection of clinically relevant effect sizes [58]. In addition, studies of RAGE isoforms in health and diseases should also ideally integrate the roles of ethnicity [59] and of RAGE gene variants [60].

This study has some limitations. First, this was a secondary analysis of a large multicenter observational study. Therefore, it may not be powered enough to avoid a type II error, and the selection of potential confounders was limited to clinical data collected by the original study. Second, it included a selected population of critically ill patients with heterogeneous ARDS risk factors, such as high-risk surgery, shock, sepsis, and pneumonia. Therefore, whether our findings could be extrapolated to a broader population of ICU patients remains unknown. Third, our exploratory study lacks a validation cohort, and future studies remain necessary to validate our findings. Fourth, the usual caveat of the precise timing between data collection and blood sampling also applies to our study, although samples obtained more than 6 hours after ICU admission were excluded from analyses. Finally, as we chose to focus only on sRAGE and esRAGE, our study does not provide insights into the influence of admission parameters on circulating levels of RAGE ligands or on other RAGE isoforms [61]. In addition, the influence of circulating levels of RAGE ligands on those of sRAGE and esRAGE was out of the scope of this study.

This study also has several strengths. First, this is to our knowledge the first report, in a large multicenter cohort of 294 patients, of the absence of significant associations between clinical/biological parameters and plasma levels of RAGE soluble forms in the setting of critical illness. Despite limitations, our findings may add novel insights into the characterization of sRAGE and esRAGE as biomarker candidates in critically ill patients, at least because no significant association was found between plasma sRAGE/esRAGE and the main baseline patient characteristics, coexisting conditions, and clinical/biological variables. Second, another strength of this study is its statistical approach that does not primarily focus on *P* values, but rather on coefficients of correlation (for quantitative variables) and effect sizes (for qualitative/categorical variables). Using multidimensional analyses, we were able to provide global and graphic views of patterns of variables that may be associated together, although the main limitation of such approach is the statistical influence of variables that are only present in a few numbers of patients (e.g., as for pancreatitis, aspiration, or treatment with corticosteroids in our cohort). However, and taken together, such findings on the (absence of) association between RAGE soluble forms and main admission parameters in critically ill patients should be useful to inform further studies of RAGE isoforms in this specific population.

In conclusion, this study is the first to investigate putative associations between plasma levels of RAGE main soluble forms (sRAGE and esRAGE) and multiple baseline parameters in a large cohort of patients at the risk of lung injury and ARDS, previously enrolled in a prospective multicenter observational study. Using multidimensional analyses, no significant association was found between plasma sRAGE or esRAGE, as measured upon ICU admission, and main patient characteristics, comorbidities, ongoing treatments, or usual clinical and biological variables. Such findings may encourage further studies of plasma levels of RAGE soluble forms as markers of acute diseases.

## Figures and Tables

**Table 1:** Main baseline characteristics at ICU admission. Data are presented as the mean ± standard deviation or *n* (%). Percentages may not exactly total 100% because of rounding. The body mass index is the weight in kilograms divided by the square of the height in meters. ICU: intensive care unit; COPD: chronic obstructive pulmonary disease; ARDS: acute respiratory distress syndrome; SAPS II: simplified acute physiology score II.

Age (years)	61.4 ± 16
Male sex	195 (66)
Body mass index (kg·m^−2^)	26.4 ± 7.2
*Primary reason for ICU admission*	
Medical	181 (62)
Emergent surgery	57 (19)
Planned surgery	50 (17)
*Coexisting chronic conditions*	
Hypertension	117 (40)
Current smoking	72 (24)
Dyslipidemia	61 (21)
Atherosclerosis	54 (18)
Diabetes	53 (18)
Cancer	48 (16)
COPD	30 (10)
Liver cirrhosis	18 (6)
Chronic renal failure requiring dialysis	9 (3)
Asthma	5 (2)
*Diagnosis at admission*	
Septic shock	33 (11)
Coma	33 (11)
Pneumonia	26 (9)
Brain trauma	24 (8)
Intra-abdominal sepsis	19 (6)
Subarachnoid hemorrhage	19 (6)
Stroke	19 (6)
Acute liver failure	18 (6)
Abdominal surgery	13 (4)
Gastrointestinal hemorrhage	13 (4)
Acute kidney injury	13 (4)
Status epilepticus	13 (4)
Thoracic trauma	11 (4)
Vascular surgery	9 (3)
Neurosurgery	9 (3)
Pancreatitis	7 (2)
Aspiration	6 (2)
Pleural effusion	4 (1)
Thoracic surgery	3 (1)
Acute severe asthma	3 (1)
Cardiac surgery	1 (0)
Kidney transplantation	1 (0)
Kidney trauma	1 (0)
Meningoencephalitis	2 (1)
*Primary ARDS risk factor*	
High-risk surgery	108 (37)
Shock	83 (28)
Sepsis	67 (23)
Pneumonia	37 (13)
Severe trauma	30 (10)
Drug overdose	9 (3)
Pancreatitis	8 (3)
Aspiration	5 (2)
Lung injury prediction score (LIPS)	5 ± 2.5
SAPS II	44.3 ± 19.7
*Specific treatment at admission*	
Vasopressor use	62 (21)
Corticosteroids	16 (5)
Neuromuscular blocking agent	2 (1)
*Ventilation at admission*	
Spontaneous breathing	150 (51)
Invasive ventilation	126 (43)
Noninvasive ventilation	18 (6)

**Table 2:** Clinical, respiratory, and biological parameters at ICU admission. Data are presented as median [interquartile range]. ICU: intensive care unit; PBW: predicted body weight; FiO_2_: fraction of inspired oxygen; PaO_2_: arterial partial pressure of oxygen; PaCO_2_: arterial partial pressure of carbon dioxide; CRP: C-reactive protein. PINI: prognostic nutritional and inflammatory index; RAGE: receptor for advanced glycation end products; sRAGE: soluble RAGE; esRAGE: endogenous secretory RAGE.

*Clinical parameters*	
Heart rate (bpm)	86.5 [70.3–104]
Mean arterial pressure (mmHg)	78 [69–88]
Respiratory rate (cpm)	17 [14–21]
Temperature (°C)	37.0 [36.5–37.4]
Glasgow coma scale	10.5 [0–15]
*Respiratory parameters*	
Tidal volume (mL)	472.5 [440.5–510]
Tidal volume (mL·kg^−1^ of PBW)	7.4 [6.7–8.1]
Positive end-expiratory pressure (cmH_2_O)	7 [6–8]
Inspiratory plateau pressure (cmH_2_O)	17 [14–20]
FiO_2_ (%)	36 [28–50]
PaO_2_ (mmHg)	92 [77–132.8]
PaCO_2_ (mmHg)	37 [32–43.8]
pH	7.4 [7.3–7.4]
Serum bicarbonate (mmol·L^−1^)	21 [18–24]
PaO_2_/FiO_2_	250 [154.3–300]
*Biological parameters*	
Serum creatinine (*μ*mol·L^−1^)	76 [56–115]
Serum urea (mmol·L^−1^)	6.6 [4.3–10]
Serum sodium (mmol·L^−1^)	139.5 [136–142]
Serum potassium (mmol·L^−1^)	3.9 [3.6–4.2]
Blood leucocytes (G·L^−1^)	11.6 [8.1–16.6]
Blood platelets (G·L^−1^)	185 [113–247]
CRP (mg·L^−1^)	108 [35.5–182]
Serum lactate (mmol·L^−1^)	1.6 [0.9–2.8]
Serum orosomucoid (g·L^−1^)	1.3 [0.9–1.9]
Serum prealbumin (g·L^−1^)	0.1 [0.1–0.2]
Serum albumin (g·L^−1^)	26.1 [22–30.9]
PINI	36.8 [8.6–167.9]
*RAGE soluble forms*	
sRAGE (pg·mL^−1^)	1006 [638–1993]
esRAGE (pg·mL^−1^)	470 [202–1000]

## Data Availability

The data used to support the findings of this study are available from the corresponding author upon request.
